# Closing the net on retroviruses

**DOI:** 10.7554/eLife.18243

**Published:** 2016-07-07

**Authors:** Jeremy Luban

**Affiliations:** Program in Molecular Medicine, University of Massachusetts Medical School, Worcester, United Statesjeremy.luban@umassmed.edu

**Keywords:** TRIM5, HIV, restriction factor, higher-order assembly, capsid, protein structure, Human, Rhesus macaque, Virus

## Abstract

Structural studies reveal how an antiviral factor forms a molecular net to restrict retroviruses including HIV-1.

**Related research articles** Li YL, Chandrasekaran V, Carter SD, Woodward CL, Christensen DE, Dryden KA, Pornillos O, Yeager M, Ganser-Pornillos BK, Jensen GJ, Sundquist WI. 2016. Primate TRIM5 proteins form hexagonal nets on HIV-1 capsids. *eLife* **5**:e16269. doi: 10.7554/eLife.16269

Wagner JM, Roganowicz MD, Skorupka K, Alam SL, Christensen DE, Doss GL, Wan Y, Frank GA, Ganser-Pornillos BK, Sundquist WI, Pornillos O. 2016. Mechanism of B-box 2 domain-mediated higher-order assembly of the retroviral restriction factor TRIM5α. *eLife* **5**:e16309. doi: 10.7554/eLife.16309

**Image** Electron cryotomography shows how TRIM5 forms a hexagonal lattice

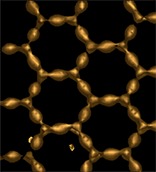

HIV-1 like other retroviruses stores its genetic material as RNA, and then converts it to DNA once inside a susceptible host cell. The process of converting RNA to DNA, which is called reverse transcription, takes place inside a protein-based shell called the capsid. The viral DNA is then integrated into the DNA of the host, where it can persist indefinitely.

Host species protect themselves against retroviruses in various ways. TRIM5, for example, is a protein that recognizes the capsid and as a result inhibits reverse transcription (Sayah et al., 2004; Stremlau et al., 2004). Recognition of the capsid lattice by TRIM5 also activates an innate immune response against the virus (Pertel et al., 2011).

TRIM5 belongs to a large family of proteins that have a RING domain, a B-box domain and a coiled-coil domain (Figure 1A). Each of these domains helps the protein to restrict the life cycle of retroviruses (Grütter and Luban, 2012). Moreover, the C-terminal half of TRIM5 binds directly to the capsid (Sebastian and Luban, 2005; Stremlau et al., 2006), and TRIM5 proteins from different species can restrict a range of retroviruses with very different capsids. Now, in two papers in eLife, researchers from the University of Virginia, the University of Utah, Caltech and Ben-Gurion University report new structural insights into how TRIM5 recognizes retroviruses with such diverse capsids (Li et al., 2016; Wagner et al., 2016).

**Figure 1.**
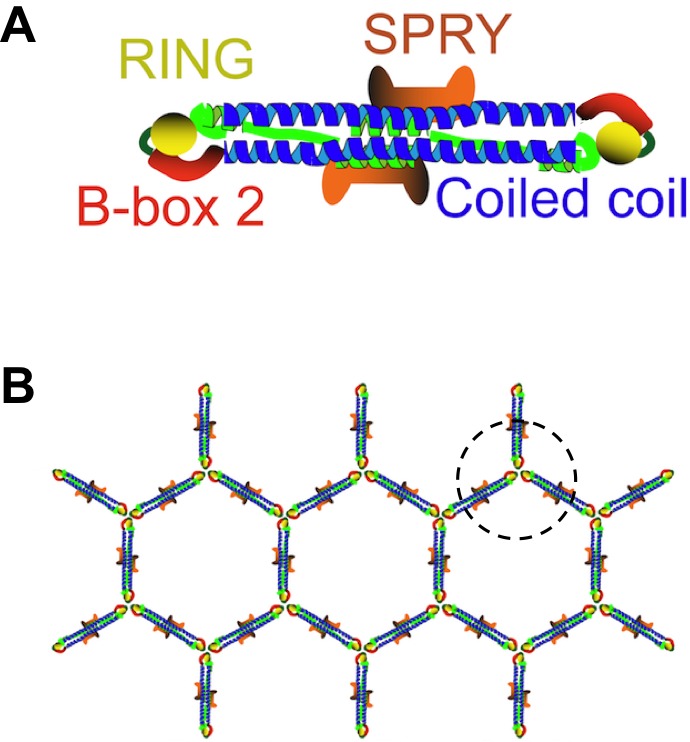
TRIM5 proteins form dimers and a hexagonal lattice. (**A**) Most TRIM5 proteins in solution will pair off to form an anti-parallel dimer via their coiled-coil domains (blue). The RING domain (yellow) and B-box domain (red) are at the ends of the dimer, and the C-terminal SPRY domains (orange) are in the center of the dimer. (**B**) When the SPRY domains bind to the capsid of a retrovirus, the B-box domains of three TRIM5 proteins associate as a trimer (circled). This, in turn, forms a hexagonal lattice of TRIM5 dimers, with the SPRY domains facing into the capsid and the RING domains pointing outwards. Flexibility in the junction between the B-box and coiled-coil domains permits TRIM5 to associate with a wide range of retroviral capsids. Adapted from Figure 1 of Li et al.

TRIM proteins are known to pair together to form dimers via their coiled-coil domains, and these dimers can make a hexagonal lattice that matches the hexagonal lattices found in retroviral capsids (Ganser-Pornillos et al., 2011; Figure 1B). However, these previous studies used artificial TRIM proteins, instead of the naturally occurring TRIM5 protein, because the native protein behaves poorly *in vitro.* For the same reason, fragments of TRIM proteins were used instead of the full-length version in other experiments to demonstrate that TRIM proteins form anti-parallel dimers rather than parallel ones (Goldstone et al., 2014; Sanchez et al., 2014; Weinert et al., 2015).

In the first paper, Barbie Ganser-Pornillos, Grant Jensen, Wesley Sundquist and colleagues – including Yen-Li Li and Viswanathan Chandrasekaran as joint first authors – report methods that can overcome the problems of working with native TRIM5 protein (Li et al., 2016). Briefly, a flat sheet of capsid lattice (which mimics the capsid of HIV-1) was used to trigger TRIM5 to form its hexagonal lattice under conditions that would otherwise prevent the TRIM5 from doing this spontaneously. Li et al. also showed that this method only worked if the TRIM5 protein could recognize and restrict the viral capsid used in the experiment. The fact that the TRIM5 lattices only form under very specific conditions *in vitro* suggests that the structures observed are relevant to what happens *in vivo*.

Li et al. went on to generate stable capsid cores, rather than flat capsid sheets. When they incubated these cores with TRIM5 protein, they saw (via electron cryotomography) that the capsid cores were decorated with hexagonal nets of TRIM5. This demonstrates that native TRIM5 protein forms a hexagonal lattice that matches the lattice of *bona fide* capsid cores.

In the second paper, Owen Pornillos and colleagues – including Jonathan Wagner as first author – report how the B-box domain of TRIM5 promotes the formation of the hexagonal lattice (Wagner et al., 2016). Crystal structures of the full-length TRIM5 protein have eluded investigators for at least 12 years. However, Wagner et al. generated a B-box domain with a shortened coiled-coil domain and a short linker shaped like a hairpin. They then used this “mini-TRIM” to grow protein crystals, but only after they had confirmed that mini-TRIM behaved like the full-length protein in a number of assays.

The crystal structures showed that the mini-TRIM proteins form both dimers and trimers via their B-box domains (Wagner et al., 2016). This mirrors the observations of another group (Keown et al., 2016). The trimers appear to link TRIM5 into a hexagonal net, which is like the net observed by electron cryotomography (Figure 1B). Wagner et al. showed that the coiled-coil domain can move in relation to the B-box domain; this flexibility could partly explain how a TRIM5 protein from a given species can recognize a diversity of capsid lattice structures.

Many questions remain regarding how the structure of TRIM5 relates to its function. For example, most of the mini-TRIM crystals formed dimers via their B-box domains; do these dimers play a functional role *in vivo*? Moreover, the antiviral activity of TRIM5 depends on its RING domain and its activity as an E3 ubiquitin ligase (Pertel et al., 2011), but how does recognizing the capsid activate this? Is this activity regulated by the B-box domain and, if so, how? Whatever the case, all the data suggest that the antiviral response of TRIM5 is activated by, and is compatible with, TRIM5 forming trimers and a hexagonal, net-like lattice.
